# Proline Dehydrogenase and Pyrroline 5 Carboxylate Dehydrogenase from *Mycobacterium tuberculosis:* Evidence for Substrate Channeling

**DOI:** 10.3390/pathogens12091171

**Published:** 2023-09-18

**Authors:** Santosh Kumar, Steven Sega, Jamie K. Lynn-Barbe, Dannika L. Harris, Jordan T. Koehn, Debbie C. Crans, Dean C. Crick

**Affiliations:** 1 Mycobacteria Research Laboratories, Department of Microbiology, Immunology and Pathology, Colorado State University, Fort Collins, CO 80523-1682, USA; santosh.kumar@uth.tmc.edu (S.K.);; 2 Department of Chemistry, University of North Carolina, Chapel Hill, NC 27599-3290, USA; jordan_koehn@med.unc.edu; 3 Chemistry Department, Colorado State University, Fort Collins, CO 80523-1682, USA; debbie.crans@colostate.edu

**Keywords:** proline catabolism, coupled assay, menaquinone, ubiquinone, lipoquinone

## Abstract

In *Mycobacterium tuberculosis*, proline dehydrogenase (PruB) and ∆^1^-pyrroline-5-carboxylate (P5C) dehydrogenase (PruA) are monofunctional enzymes that catalyze proline oxidation to glutamate via the intermediates P5C and L-glutamate-γ-semialdehyde. Both enzymes are essential for the replication of pathogenic *M. tuberculosis*. Highly active enzymes were expressed and purified using a *Mycobacterium smegmatis* expression system. The purified enzymes were characterized using natural substrates and chemically synthesized analogs. The structural requirements of the quinone electron acceptor were examined. PruB displayed activity with all tested lipoquinone analogs (naphthoquinone or benzoquinone). In PruB assays utilizing analogs of the native naphthoquinone [MK-9 (II-H_2_)] specificity constants *K_cat_/K_m_* were an order of magnitude greater for the menaquinone analogs than the benzoquinone analogs. In addition, mycobacterial PruA was enzymatically characterized for the first time using exogenous chemically synthesized P5C. A *K_m_* value of 120 ± 0.015 µM was determined for P5C, while the *K_m_* value for NAD^+^ was shown to be 33 ± 4.3 µM. Furthermore, proline competitively inhibited PruA activity and coupled enzyme assays, suggesting that the recombinant purified monofunctional PruB and PruA enzymes of *M. tuberculosis* channel substrate likely increase metabolic flux and protect the bacterium from methylglyoxal toxicity.

## 1. Introduction

The enzymes involved in the oxidation of proline to glutamate play important roles in the physiology and metabolism of *Mycobacterium tuberculosis*, which is the etiological agent of tuberculosis. Genes involved in proline metabolism are strongly expressed during nutrient starvation or hypoxia [1], and proline metabolism has been implicated in mycobacterial infections [2,3]. In addition, the enzymes involved in the oxidation of proline to glutamate are important for achieving *M. tuberculosis* growth [1,4,5,6]. Therefore, there is interest in using these enzymes as potential drug targets [7,8].

**Figure 1:**
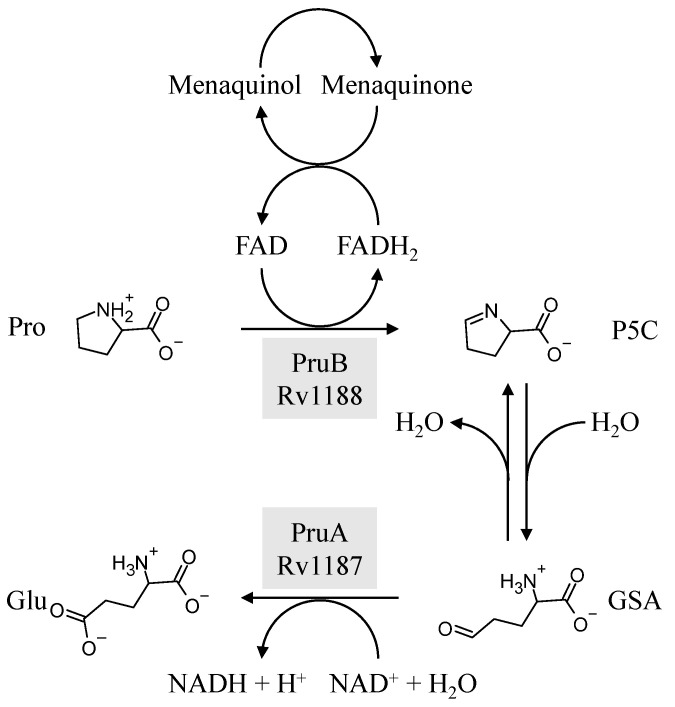
Reactions catalyzed by *M. tuberculosis* PruB and PruA enzymes. For PruB, proline dehydrogenase catalyzes the formation of ∆^1^-pyrroline-5-carboxylic acid (P5C), which is in equilibrium with glutamate-γ-semialdehyde (GSA), as well as the reduction in menaquinone. P5C is further oxidized by PruA to generate glutamate with the concomitant reduction in NAD^+^.

Proline oxidation involves the conversion of proline to ∆^1^-pyrroline-5-carboxylic acid (P5C, Figure 1) via catalyzation by proline dehydrogenase, using FAD as a cofactor. P5C exists in tautomeric equilibrium with glutamate-semialdehyde (GSA), which is converted into glutamate by a NAD^+^-dependent ∆^1^-pyrroline-5-carboxylate dehydrogenase. However, the tertiary/quaternary structures of the enzymes catalyzing these reactions differ from organism to organism [9]. Some bacterial species possess a bifunctional enzyme that accomplishes the conversion of proline into glutamate [10], while other bacteria have a trifunctional enzyme in which an additional DNA-binding domain acts as a transcriptional regulator, as seen in *Escherichia coli* PutA [11]. However, all eukaryotes and some bacteria, including *M. tuberculosis* [1,4,7,8], are restricted to pairs of monofunctional enzymes [12,13]. In mycobacteria, these enzymes are encoded by *pruA* and *pruB* and *rv1187* and *rv1188*, respectively.

Mycobacterial PruB has previously been cloned, purified, and characterized using the artificial electron acceptor 2,6-dichlorophenolindophenol (DCPIP) [8]. Steady-state kinetic analysis revealed a ping-pong mechanism, and site-directed mutagenesis suggested that Lys110 is involved in the catalytic mechanism [8]. Mycobacterial PruA has also been cloned and purified, and the crystal structure has been determined [7]. However, due to a lack of exogenous substrate(s) for PruA, [P5C/GSA (Figure 1)] and aggregation of recombinant PruB, these researchers were unable to conduct a kinetic analysis, although they were able to demonstrate that both PruB and PruA were required to convert proline to glutamate in assays containing both recombinant proteins.

In *E. coli* and *Staphylococcus typhimurium*, it has been shown that there is direct transfer of P5C between the proline dehydrogenase and P5C dehydrogenase active sites of the multifunctional enzyme PutA. This substrate channeling between proline dehydrogenase and P5C dehydrogenase active sites has been extensively studied [14,15]. Possible reasons for channeling P5C/GSA may include limiting interactions with other molecules and regulating signaling activity, as P5C and GSA have been shown to react with metabolites [1,16], inhibit enzymes [17], and affect signaling pathways [18]. The Rosetta stone hypothesis suggests that if two or more enzyme activities are fused in a single protein in one organism, as exemplified by PutA in *E. coli* [14], it is true that in an organism in which the activities are not fused into a single protein, the individual proteins constituting the same activities may interact [19,20]. Although mycobacterial PruB and PruA mixtures were previously shown to be active, it was not possible to demonstrate evidence of stable complex formation between the enzymes; however, such an interaction seemed to be possible, as the PruA active site is solvent accessible via a channel that is potentially suitable for substrate channeling [7]. Thus, the question of whether non-covalently linked PruB and PruA in *M. tuberculosis* utilize a substrate channeling mechanism like that seen in PutA remains unanswered. 

Here, we report the kinetic characterization of PruA from *M. tuberculosis*, discuss the kinetic characterization of mycobacterial PruB utilizing natural lipoquinone electron acceptor analogs, and provide enzymatic evidence of substrate channeling between a monofunctional proline dehydrogenase (PruB) and a ∆^1^-pyrroline- 5-carboxylate dehydrogenase (PruA).

## 2. Materials and Methods

### 2.1. Materials 

High Fidelity Taq DNA polymerase was acquired from Roche Diagnostics (South San Francisco, CA, USA), and Pierce BCA Protein Assay Kits were purchased from ThermoFisher Scientific (Waltham, MA, USA). Oligonucleotide primers were synthesized via Integrated DNA Technologies. Plasmid mini-prep and PCR purification kits were acquired from ThermoFisher Scientific (Waltham, MA, USA). Hygromycin B was acquired from Calbiochem. Bacterial media and growth supplements (7H9, 7H10, ADC, OADC, and LB) were acquired from BD Difco. Vitamin K2 (MK-4), UQ-1, UQ-2, NAD^+^, FAD, 2-aminobenzaldehyde, goat antimouse IgG alkaline phosphatase conjugated secondary antibody, BCIP/NBT tablets, acetamide, Complete Protease Inhibitor Cocktail, Ni-NTA beads, Tween-80, and Triton X-100 were purchased from Millipore Sigma (Burlington, MA, USA). 6×-His tag antibodies were acquired from ThermoFisher Scientific (Waltham, MA, USA). MK-1, MK-1(H_2_), and MK-2 were prepared as previously described [21,22,23,24] (see Table 1 to review the structures of the various lipoquinone molecules described in this study).

### 2.2. Cloning, Expression, and Purification of PruB and PruA 

PruB and PruA were amplified from *M. tuberculosis* genomic DNA using the primers listed in Table S1. Amplicons were initially cloned in the pET28a^+^ vector; however, due to a lack of protein expression in *E. coli*, both genes were further subcloned in the pMyNT mycobacterial expression vector [25,26]. The fidelity of the clones was verified via restriction digestion and DNA sequencing. The recombinant plasmids were used to transform *Mycobacterium smegmatis.* Transformants were inoculated in 10 ml of 7H9 Middlebrook Medium supplemented with 0.2% (*v*/*v*) glycerol, 10% ADC, 0.05% Tween-80, and 50 µg/mL of hygromycin B, and they were incubated at 37 °C for 2 days. The culture was diluted into 1000 mL of Middlebrook 7H9 medium and incubated at 37 °C. At the mid-log phase, bacteria protein expression was induced with 0.2% (*w*/*v*) acetamide Millipore Sigma (Burlington, MA, USA) for another 24–36 h at 37 °C, and cells were harvested via centrifugation. Harvested cells were resuspended in buffer containing 150 mM of Tris-HCl (pH 7.0), 100 mM of NaCl, and 1 mM of phenylmethylsulfonyl fluoride and protease inhibitor cocktail. 

The harvested bacteria were broken via sonication in buffer containing 0.1% Triton X-100. The detergent solubilized homogenate was obtained via centrifugation at 27,000× *g* for 30 min at 4 °C. This fraction was then incubated at 4 °C with Ni-NTA beads (Burlington, MA, USA) [27]. Bound protein was eluted with buffer containing 150 mM of Tris-HCl (pH 7.0), 100 mM of NaCl, and 250 mM of imidazole. Eluted protein was further purified using PD-10 columns and concentrated via ultra-filtration in 150 mM of Tris-HCl containing 100 mM of NaCl. The purity of the protein was assessed using 12% SDS-PAGE. Purification yielded ~1 mg of protein per liter of culture volume. The protein concentration was determined using a BCA Protein Assay Kit, glycerol was added up to 10%, and protein was stored in aliquots at −80 °C. 

PruA protein was isolated from the soluble fraction with the help of a Ni-NTA affinity column. Eluted protein was further purified using PD-10 columns and concentrated via ultra-filtration, as described above.

### 2.3. Measurement of PruB Enzymatic Activity

The reaction mixtures contained 20 mM of Tris-HCl (pH 7.0), PruB, lipoquinone, and L-proline in a total volume of 200 µL at 25 °C in the presence of 5 µM of FAD. The enzyme activity was spectrophotometrically measured following the decrease in the absorbance at 270 (MK) or 278 (UQ) nm. In all cases in which menaquinone was used as the electron acceptor, the lipoquinone was solubilized in 20% Tween-80, which was diluted to a final concentration of 0.5% in the assay mixtures. 

### 2.4. Synthesis of DL-Pyrroline-5-Carboxylic Acid [(DL)-P5C]

(DL)-5PC (50/50 mixture) was synthesized as previously described [28]. In brief, a stir bar was added to a 250-milliliter round-bottom flask containing DL-hydroxylysine (2.0 mmol) and double distilled H_2_O (28 mL), which was cooled to 0 °C in an ice-H_2_O bath. Then, 50 mM of sodium metaperiodate (44 mL, which was adjusted to pH 7.0 using a glass electrode and a small volume of 1 M of NaOH in dim light), which had been cooled to 0 °C on ice for ~10 min. was quickly added. The reaction mixture was covered in aluminum foil and stirred for 8 min at 0 °C. The periodate solution was quenched with 1-molarity glycerol and stirred for 2 min at 0 °C. Then, the solution was acidified with 6-molarity HCl. The resulting cold reaction mixture was then poured onto a Dowex 50 column (2 × 62 cm), and ~30 mL of flow through was collected and set aside at ~4 °C, as P5C is stable in 1-molarity HCl at 4 °C [28]. The column was then moved to room temperature for the remainder of the procedure, and (DL)-P5C elution was accomplished with 1.0-molarity HCl at a flow rate of ~1 mL/min; fractions were immediately collected (8.5-milliliter fraction volume, with 70 fractions being collected). Fractions containing (DL)-5PC (50/50 mixture) were stored at 4 °C and neutralized on the day of experiments on ice with 10-molarity NaOH [28]. 

### 2.5. Measurement of PruA Enzymatic Activity

P5C dehydrogenase activity was measured by monitoring the initial velocity of NADH formation at 340 nm. Assays were performed using exogenous (DL)-P5C and recombinant PruA in 20 mM of Tris-HCl (pH 7.0). (DL)-P5C was neutralized with 10-molarity NaOH on ice immediately prior to the performance of assays. The kinetic parameters for P5C were determined by varying the P5C/GSA concentration (0.01–2.0 mM) while holding the NAD^+^ concentration constant at 0.2 mM in 20 mM of Tris-HCl (pH 7.0). The *K_m_* and *K_cat_* values for NAD^+^ were determined by varying the NAD^+^ concentration (0–400 µM) while holding the (DL)-P5C concentration constant (300 μM). All assays were performed in 200-microliter reaction volumes. The inhibition of PruA activity by proline was determined by varying (DL)-P5C (1–300 µM) at the indicated concentrations of proline (0–32 mM). Initial velocities were fit to a competitive inhibition equation.

(1) $$\frac{V_{max}}{\left[1+\left(K_{m}/S\right)\times \left(1+I/K_{I}\right)\right]}$$

using SigmaPlot 14 Enzyme Kinetics Module (Systat Inc., Richmond, CA, USA).

### 2.6. Coupled PruB-PruA Reaction

The coupled *M. tuberculosis* proline dehydrogenase-P5C dehydrogenase activity was monitored by following the NADH absorbance at 340 nm or fluorescence (excitation at 340 nm and monitoring fluorescence emission at 460 nm). Assays were performed at 25 °C in 20 mM of Tris-HCl (pH 7.0) containing 100 µM of UQ-1, 0.2 mM of NAD^+^, 40 mM of proline, and 0.5 μM of PruB enzyme over a 5-minute incubation period. The reaction was initiated via the addition of 0.5 μM of PruA enzyme, and the production of NADH was followed over 30 min.

### 2.7. P5C Trapping Assays

Trapping assays were performed as described, with coupled assays performed with the addition of 1mM of *o*-aminobenzaldehyde (*o*-AB). In the presence of P5C, *o*-AB rapidly formed a dihydroquinazolinium compound that could be monitored spectrophotometrically at 443 nm [29].

## 3. Results and Discussion

### 3.1. Expression and Purification of PruB and PruA

Both *pruB* and *pruA* genes were amplified from genomic DNA of *M. tuberculosis* and cloned in the pET28a^+^ vector. Due to the lack of protein expression in *E. coli*, both genes were further sub-cloned in pMyNT, which is a mycobacterial expression vector [21,25]. Although the protein is associated with membrane-enriched preparations, the His-tagged recombinant PruB could be purified to at least 80% purity, as estimated via SDS-PAGE analysis, after solubilization in buffer containing 0.1% Triton X-100 (Figure S1). In contrast, PruA was purified from the soluble cytosol fraction [27]. Both nickel-NTA-purified proteins had appropriate apparent molecular weight, as determined via SDS-PAGE and Western blot analyses. The purified recombinant PruB protein was yellow in color, which was in keeping with the observation that it has a predicted FAD-binding domain and suggests that the protein is co-purified with bound FAD.

### 3.2. Biochemical Characterization of PruB

Enzyme assays were conducted at 25 °C and pH 7.0 in 20 mM of Tris-HCl. Commercially available MK-4, which is a naphthoquinone with four isoprene units (Table 1), was initially used as a prospective electron acceptor for PruB; however, no enzyme activity could be detected. MK has very limited solubility in aqueous solution, the calculated logP (ClogP) value of MK-4 is 10.9, and the compound is sensitive to light exposure [30]. The resulting technical issues forced the use of chemically synthesized MK-1 and MK-2, which share the same naphthoquinone head group as the natural lipoquinone found in *M. tuberculosis* membranes, being the electron acceptor in the assays (Table 1). These molecules with ClogP values of 4.8 and 5.0, respectively, were acceptable substrates for PruB (see Figure S2 for representative UV-vis traces of the kinetic assay). However, all menaquinone derivatives utilized required 20% Tween-80 to perform solubilization of the stock material in aqueous buffer, which was further diluted to the 0.5% utilized in enzyme assays. Additionally, water-soluble commercially available analogs of ubiquinone (UQ-1 and UQ-2, Table 1) were utilized as substrates. PruB oxidoreductase activities were assayed by measuring the reduction in UQ or MK. The reaction mixtures contained 20 mM of Tris-HCl, PruB, lipoquinone, FAD, and L-proline at a total volume of 200 µL. The enzyme activity was spectrophotometrically measured following the decrease in the absorbance at 270 (MK) or 278 (UQ) nm. However, as mycobacterial PruB enzymes require FAD as a cofactor, and FAD reoxidation appears to be the rate-limiting step in the overall reaction [8], the addition of FAD to the assay buffer resulted in little difference in activity, confirming the copurification of the protein and co-factor.

Among the UQ analogs tested, UQ-2 was found to show the least activity. MK-1 was found to support more activity than MK-2, and no activity could be detected using MK-4. The lack of activity in the presence of MK-4 is attributed to the lack of sufficient solubility under the tested conditions. PruB enzyme activity was also detected with MK-1(H_2_), which is a naphthoquinone analogue with a single saturated isoprene unit.

### 3.3. Kinetic Parameters for PruB

**Figure 2:**
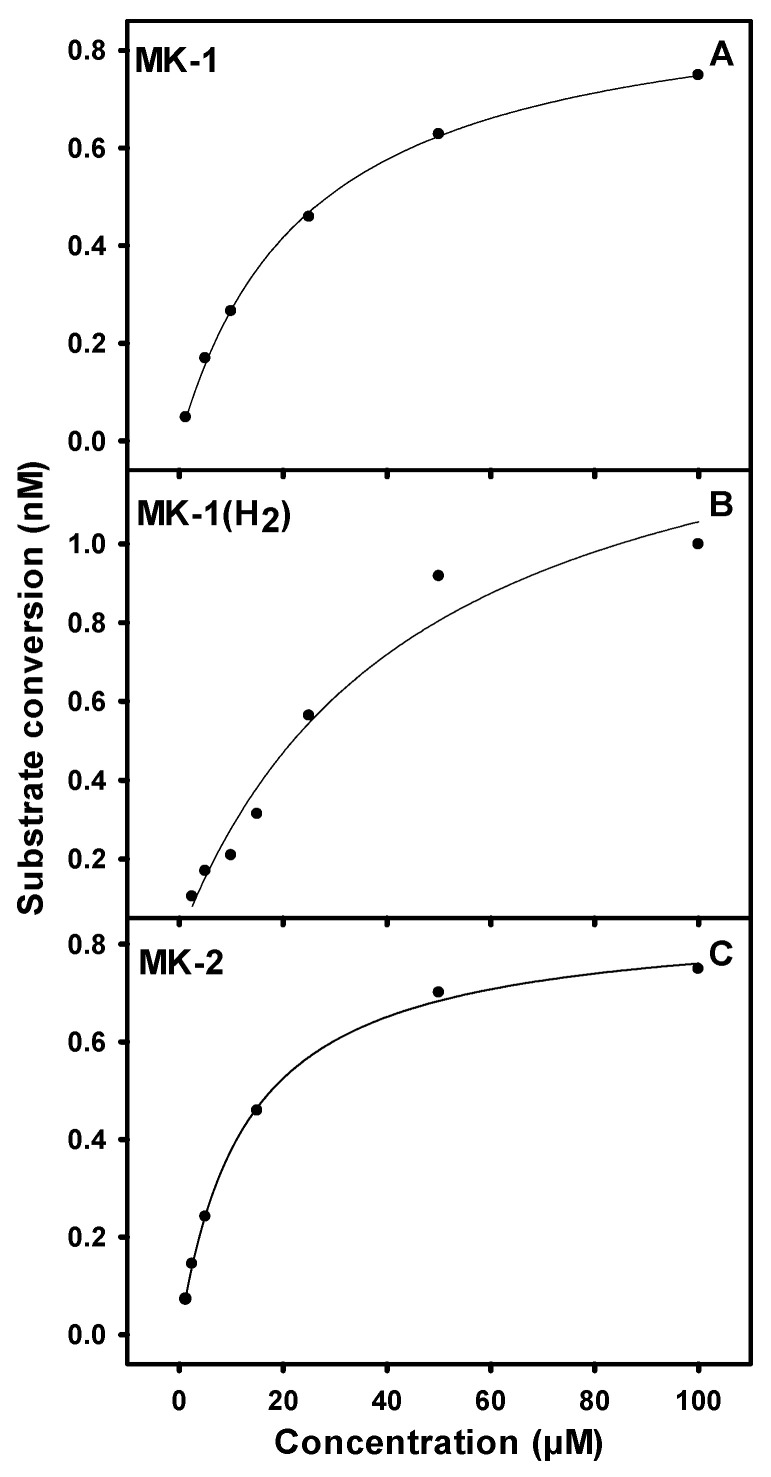
Representative Michaelis–Menten curves for PruB showing the effects of MK-1 (Panel (**A**)), MK-1(H_2_) (Panel (**B**)), or MK-2 (Panel (**C**)) in the presence of saturating concentrations of proline (20mM). Assays contained 25 ng of PruB in 200 µL of 20mM of Tris-HCl at pH 7.0 and were incubated at 25 °C for 30 min. Activities were monitored following the decrease in the absorbance at 270 nm. Calculated kinetic parameters can be found in Table 2.

The maximum enzyme activity was found at 25 °C and pH 7.0 [8]. The addition of CHAPS, cholate, or Tween-80 at 0.1 to 0.2% had no significant effect on activity; however, the addition of CHAPS or cholate at 0.5% or greater reduced activity by 90%. The addition of Tween-80 at 0.5% reduced the enzyme activity by 20% but was required to solubilize the naphthoquinones. The kinetic parameters for PruB of *M. tuberculosis* were determined using the Michaelis–Menten kinetic assumptions, as shown in Figure 2 and Figure S3. The PruB *K_m_* value for UQ-1 (*K_m_*^*UQ*-1^) in presence of FAD was found to be 9.7 µM at saturating concentrations of L-proline (Table 2). The *K_m_^proline^* was determined to be 2.5 mM in the presence of saturating levels of UQ-1, while *K_m_^proline^* at saturating concentrations of MK-1 was found to be 14 mM. The *K_m_* values of the napthoquinone analogs MK-1 and MK-2 in presence of FAD and 0.5% Tween-80 were determined to be 23 µM and 12 µM, respectively, at saturating levels of L-proline. The *K_m_* for MK-1(H_2_) was found to be 46 µM, which was the highest *K_m_* of all tested quinone analogs. The calculated parameters *K_cat_* and *K_cat_/K_m_* are presented in Table 2.

*K_m_*^*UQ*-1^ in presence of exogenous FAD and saturating L-proline were found to be 9.6 µM, which is 10 times less than that reported for the bifunctional *E. coli* PutA [31]. In addition, the *K_m_^proline^* in the presence of saturating UQ-1 was found to be 2.5 mM, which is 18-fold less than that reported for *E. coli* PutA [31]. However, it must be kept in mind that these values reflect the half reactions catalyzed by the bifunctional enzymes in comparison to the purified recombinant proline dehydrogenase derived from *M. tuberculosis*. The *K_m_*^*proline*^ reported here is similar to previously reported for PruB (5.7 mM) in the presence of saturating DCPIP as the final electron acceptor [8].

The *K_m_* values of the natural naphthoquinone substrate analogs MK-1 and MK-2 were found to be 23 µM and 12 µM, respectively. Interestingly, PruB can utilize benzoquinones as electron acceptors as efficiently as the naphthoquinones; the calculated *K_cat_* values were similar for MK-1, MK-2, and UQ-1, suggesting that PruB does not differentiate between these molecules. Additionally, PruB is able to utilize MK-1(H_2_) as an electron acceptor, indicating that a double bond in the isoprene side chain is not essential for PruB activity. 

### 3.4. Characterization and Kinetic Parameters of PruA

**Figure 3:**
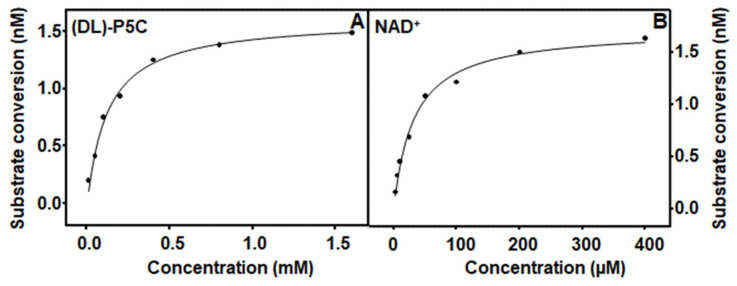
Representative Michaelis–Menten curves for PruA showing the effects of the P5C concentration in an assay at a 0.2-millimolar NAD^+^ (Panel (**A**)) concentration and a 0.3-millimolar P5C (Panel (**B**)) concentration. Assays contained 500 ng of PruA in 200 µL of 20 mM of Tris-HCl at pH 7.0 and were incubated at 25 °C for 30 min. Activities were monitored following the increase in the absorbance at 340 nm. The calculated kinetic parameters can be found in Table 3.

*M. tuberculosis* PruA activity was determined using previously synthesized exogenous (DL)-P5C and 0.5 μg of the PruA enzyme. Synthesized and stored (DL)-P5C was neutralized with 10 M of NaOH immediately prior to its use in assays. Like PruB activity, PruA was optimal at 25 °C and pH 7.0 in 20 mM of Tris-HCl. Kinetic parameters for PruA were determined using Michaelis–Menten kinetic assumptions. The representative saturation curves are shown in Figure 3.

**Figure 4:**
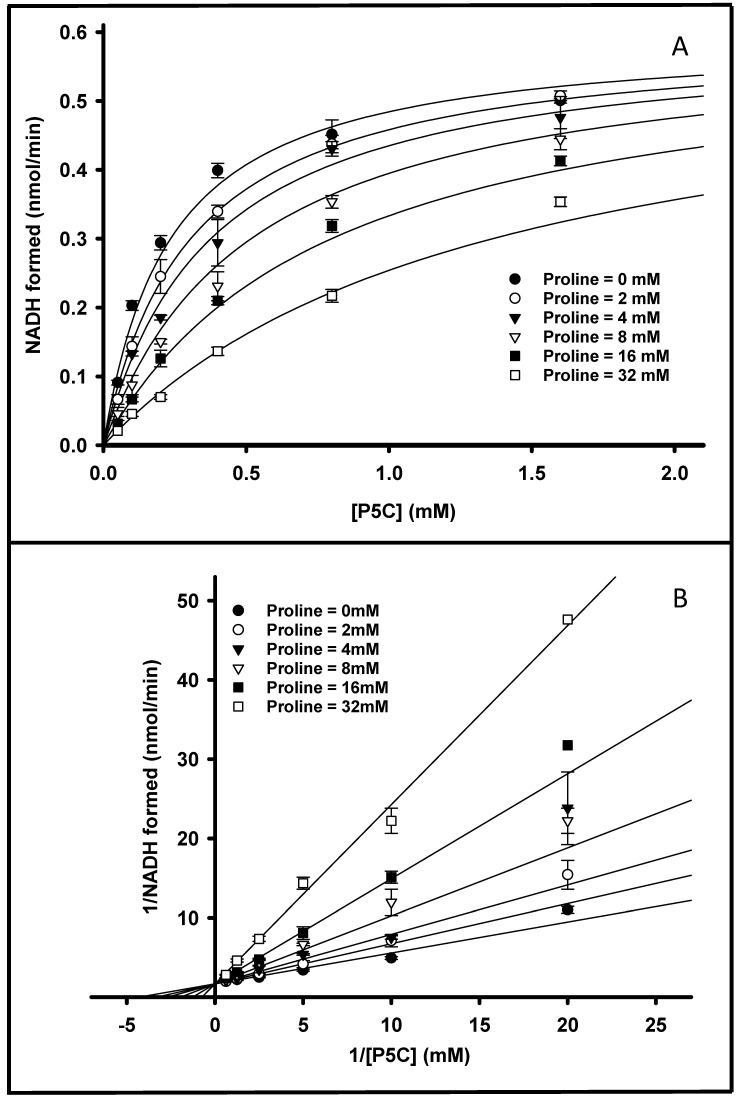
Inhibition of PruA by L-proline. Michaelis–Menten curves are shown in Panel (**A**). The proline concentration varied from 0 to 32 mM and the P5C concentration varied from 0.1 to 1.6 mM in 20 mM Tris-HCl at pH 7.0 at 25 °C for 30 min. Panel (**B**) shows the double reciprocal plots of the same data. Error bars indicate the standard deviation of the mean of three independent experiments. The *K_I_^proline^* (6.7 mM) was calculated using the fit to a competitive inhibition model (Equation (1)).

The *K_m_* for NAD^+^ was found to be 33 µM, which is two-fold less than that of the monofunctional enzyme derived from *Thermus thermophilus* [13] and ten-fold less than that of the bifunctional enzyme derived from *Bradyrhizobium japonicum* [32]. In the absence of proline, the *K_m_^P^*^5*C*^ was found to be 120 µM, as shown in Table 3, which is three times higher than that reported for the monofunctional enzyme derived from *Thermus thermophilus* [13,33]. The addition of proline to the reaction mixtures resulted in a *K_m_^P^*^5*C*^ nearly twice as high (230 μM) as that observed in the absence of proline, suggesting the potential competitive inhibition of PruA activity, an observation that is consistent with similar conclusions previously drawn for both monofunctional and bifunctional enzymes [31,33]. Subsequent experimentation showed that the addition of increasing proline concentrations to the assay mixtures resulted in a pattern of decreased PruA enzyme activity that fit the competitive inhibition model well (Figure 4 and Table S2). An inhibition constant (*K_i_*) of 6.7 mM was calculated.

### 3.5. PruB-PruA Channeling

As noted above, the central pathway of proline utilization in *M. tuberculosis* is catalyzed via the activities of two different monofunctional enzymes, as indicated in Figure 1. The addition of both PruB and PruA to the reaction mixture results in a coupled assay, which can be used to determine the rate of the conversion of proline into glutamate in the presence of NAD^+^.

A molar ratio of PruB to PruA (1:1), ensuring that the coupled reaction was not limited by the concentration of either enzyme, was determined by varying PruA (0.125–6 µM) and fixing PruB at 0.5 µM (Figure S4). The reaction mixture containing 20 mM of tris-HCl (pH 7.0), 100 µM of UQ1, 0.2 mM of NAD^+^, 40 mM of proline, and 0.5 µM of PruB was pre-incubated for 5 min. The reaction was initiated via the addition of the PruA enzyme for 30 min. The increase in absorbance or fluorescence was recorded by measuring the conversion of NAD^+^ into NADH in the PruA-catalyzed reaction. 

The effect of one enzyme on the reaction rate of the other enzyme was also monitored (Figure S5). Reaction mixtures contained 20 mM of Tris-HCl (pH 7.0), 5 µM of FAD, 100 µM of UQ-1, 0.2 mM of NAD^+^, 40 mM of proline, and equimolar concentrations (0.5 µM) of enzymes. In addition, the activity of each enzyme was separately monitored under the same conditions; no change in the activity of either enzyme was observed in the presence or absence of the other enzyme (Figure S5).

**Figure 5:**
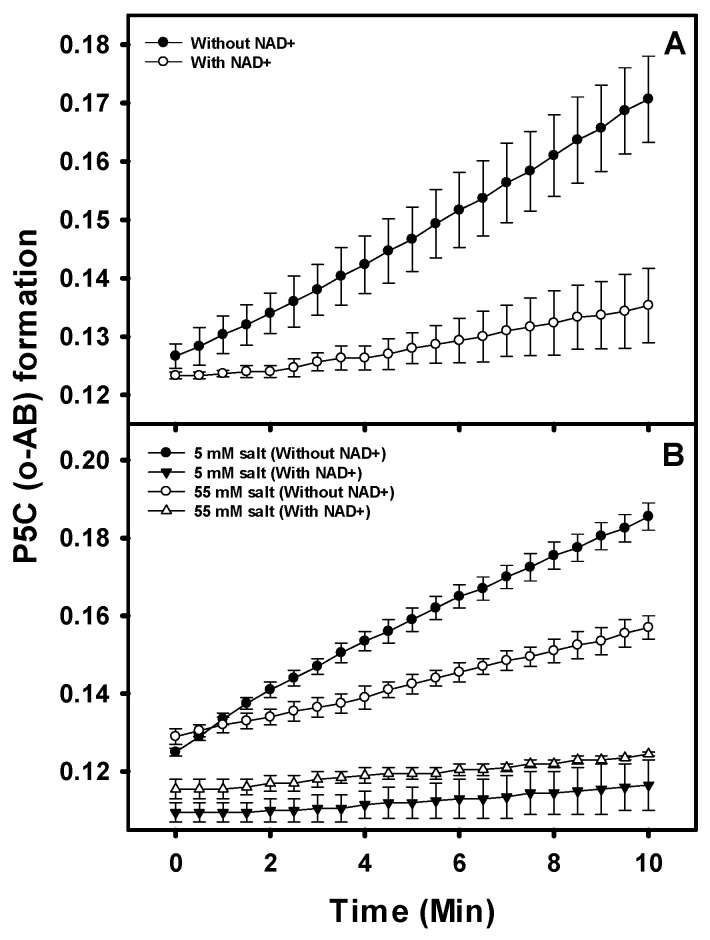
Intermediate P5C-GSA trapping with *o*-aminobenzaldehyde. P5C/GSA is bound by *o*-aminobenzaldehyde (*o*-AB), forming the dihydroquinazolinium complex that is detectable at 443 nm. The reaction mixtures contained 20 mM of Tris-HCl (pH 7.0), 100 µM of UQ1, 0.2 mM of NAD^+^, 20 mM of proline, and equimolar concentrations (0.5 µM) of PruB and PruA enzymes (Panel (**A**)). Reactions were also performed in the presence of low and high salt concentrations (Panel (**B**)). Error bars indicate the standard deviation of the mean of three independent experiments.

The compound *o*-aminobenzaldehyde (*o*-AB) reacts with free P5C in solution, forming dihydroquinazolinium that is detectable at 443 nm [28]. The addition of *o*-AB to the assay mixture results in the trapping of the P5C via rapid formation of dihydroquinazolinium as it is released from the PruB. P5C trapping was examined using equimolar mixtures of PruB and PruA in assays with and without NAD^+^. The formation of the dihydroquinazolinium was substantially reduced when NAD^+^ (200 μM) was present in the reaction mixture (Figure 5A). Thus, in the presence of NAD^+^ P5C, trapping is significantly decreased, indicating that the P5C is not accessible to the *o*-AB. These observations indicate that the bulk of P5C is channeled directly to PruA from PruB, rather than being released into the surrounding milieu. 

Additionally, similar reactions were conducted in the presence of high concentrations of salt in anticipation that the salt might disrupt any electrostatic interactions between PruB and PruA, increasing the P5C available to the *o*-AB in solution. There was no observable effect of salt addition on the activity of the individual enzymes, and increased P5C leakage at a high salt concentration was not detected (Figure 5B).

Overall, it is thought that metabolite channeling, occurring within either multifunctional enzymes or enzyme complexes, enhances metabolic flux, thereby improving the cellular fitness of the organism [34,35]. Substrate channeling has been extensively studied in various metabolic pathways, such as fatty acid β oxidation [36], the tricarboxylic acid cycle [37], and purine biosynthesis [38,39]. In the proline catabolism pathway, evidence for substrate channeling for the proline/P5C dehydrogenase reactions was reported in PutA derived from *Salmonella typhimurium* [15], *Bradyrhizobium japonicum* [32], and *Geobactor sulfurreducens* [40]; these reports have provided unprecedented molecular details of P5C/GSA channeling [32]. A novel hysteretic mechanism described for the *E. coli* PutA suggested that a channeling step in the overall proline/P5C dehydrogenase reactions was activated in the first few catalytic turnovers [14,41]. In this report, we have provided evidence of substrate channeling between non-covalently linked *M. tuberculosis* PruB and PruA enzymes, which is consistent with the Rosetta stone hypothesis [19,20].

Organisms in which proline dehydrogenase and P5C dehydrogenase are fused may have the ability to regulate P5C/GSA levels and utilize P5C as a metabolite signaling molecule or drive the proline-P5C redox cycle [18,42]. In contrast, in organisms with monofunctional proline and P5C dehydrogenases, the additional regulation of the proline catabolic pathway maybe possible due to dynamic interactions. It has been suggested that the proline oxidation protects mycobacterial cells from methylglyoxal toxicity [1]. Methylglyoxal is an abundant reactive electrophilic species formed from glucose, lipid, and protein metabolism that reacts with nucleophilic centers in macromolecules, such as DNA, RNA, and protein causing covalent glycation end products [43,44]. Although multiple methylglyoxal degradation/detoxification pathways have been reported [43], it has been demonstrated that a functional PruB but not PruA protects mycobacteria from methylglyoxal toxicity [1]. 

Methylglyoxal can react with P5C to form non-toxic 2-acetyl-1-pyrroline [45]. However, the data presented indicate that in equimolar concentrations of purified recombinant PruA and B, as well as optimized conditions including saturating NAD^+^, the bulk of P5C is converted into glutamate in vitro. However, this outcome may not be the case in vivo. Interacting monofunctional proline and P5C dehydrogenases exhibiting imperfect metabolite channeling, perhaps occurring due to allosteric or other translational/post-translational regulation, may provide an advantage in terms of cell survival. 

## Figures and Tables

**Table 1:** Structures of lipoquinones described in this study.

Lipoquinone	Structure
UQ-1	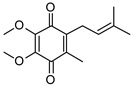
UQ-2	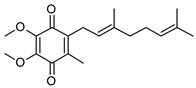
MK-1	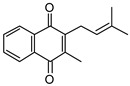
MK-1(H_2_)	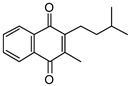
MK-2	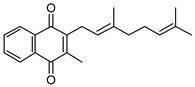
MK-4	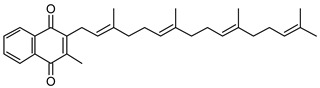

**Table 2:** Calculated kinetic parameters of proline dehydrogenase (PruB). *K_m_* and *V_max_* values of UQ-1, MK-1, MK-1(H_2_), and MK-2 were determined in the presence of saturating levels of proline, and kinetic parameters of proline were determined in the presence of UQ-1 or MK-1, as indicated. Assays were performed at 25 °C and pH 7.0 in 20 mM of Tris-HCl. PruB activity was assayed by measuring the reduction in the ubiquinone or menaquinone. Error indicates the standard deviation of the mean of the three independent experiments. nd = not determined.

Substrate	*K_m_*	*V_max_* (pmol)	*K_cat_* (S^−1^)	*K_cat_*/*K_m_* (S^−1^ M^−1^)
UQ-1	9.7 ± 1.4 μM	1.6 ± 0.39	2.3	2.3 × 10^5^
MK-1	23 ± 3.2 μM	1.9 ± 0.04	2.8	1.2 × 10^6^
MK-1(H_2_)	46 ± 13 μM	3.2 ± 0.21	4.6	1.0 × 10^6^
MK-2	12 ± 0.66 μM	1.8 ± 0.01	2.6	2.1 ×10^6^
Proline (saturating UQ-1)	2.5 ± 0.3 mM	1.2 ± 0.03	1.7	6.9 × 10^2^
Proline (saturating MK-1)	14 ± 4.1 mM	nd	nd	nd

**Table 3:** Calculated kinetic parameters of PruA. The kinetic parameters were measured in 20 mM of tris-HCl buffer (pH 7.0) at 25 °C. Substrate concentrations are described in the Methods section. The increase in the absorbance at 340 nm measures the conversion of NAD^+^ into NADH in the PruA- catalyzed reaction. Error indicates the standard deviation of the mean of the three independent experiments.

Substrate	*K_m_* (µM)	*V_max_* (pmol)	*K_cat_* (S^−1^)	*K_cat_*/*K_m_* (S^−1^ M^−1^)
NAD^+^	33 ± 4.3	0.57 ± 0.06	1.1	3.4 × 10^5^
P5C	120 ± 0.02	0.54 ± 0.05	1.1	8.5 × 10^3^

## Data Availability

Not applicable.

## Supplementary Materials

The following supporting information can be downloaded via the following link: https://www.mdpi.com/article/10.3390/pathogens12091171/s1, Figure S1. Purification of PruB and PruA. Figure S2. Representative UV-Vis traces of enzyme activity. Figure S3: Representative Michaelis–Menten curves for PruB; Figure S4: PruB-PruA coupled reaction; Figure S5: PruB and PruA do not affect the reaction rate of the other enzyme; Table S1: Primers used in the amplification of PruA and PruB constructs; Table S2: Non-linear fit of data shown in Figure 4 to a competitive inhibition model.
