# Supplementary material for: Proline Dehydrogenase and Pyrroline 5 Carboxylate Dehydrogenase from Mycobacterium tuberculosis: Evidence for Substrate Channeling
Source: Pathogens. 2023 Sep 18;12(9):1171. doi: 10.3390/pathogens12091171 (PMC10537722; doi:10.3390/pathogens12091171)
Supplement: Supplementary file 1 [file pathogens-12-01171-s001.zip › pathogens-2551185-supplementary.pdf]

Supplementary Materials

Proline Dehydrogenase and Pyrroline 5 Carboxylate Dehydrogenase from *Mycobacterium tuberculosis:* Evidence for substrate channeling

Santosh Kumar[1], Steven Sega[1], Jamie K. Lynn-Barbe[1], Dannika L. Harris[1], Jordan T. Koehn[2], Debbie C. Crans[3], and Dean C. Crick[1*]

[1]Mycobacteria Research Laboratories, Department of Microbiology, Immunology and Pathology, 1682 Campus Delivery, Fort Collins, CO 80523, USA.

[2]Department of Chemistry, University of North Carolina, Chapel Hill NC 27599-3290, USA

[3]Chemistry Department, Colorado State University, Fort Collins, Colorado 80523, United States.

*To whom correspondence should be addressed:

Mycobacteria Research Laboratories, Department of Microbiology, Immunology and Pathology, 1682 Campus Delivery, Fort Collins, CO 80523, USA.

E-mail: Dean.Crick@colostate.edu; Tel. (+1) 970 491 3308; Fax (+1) 970 491 1815

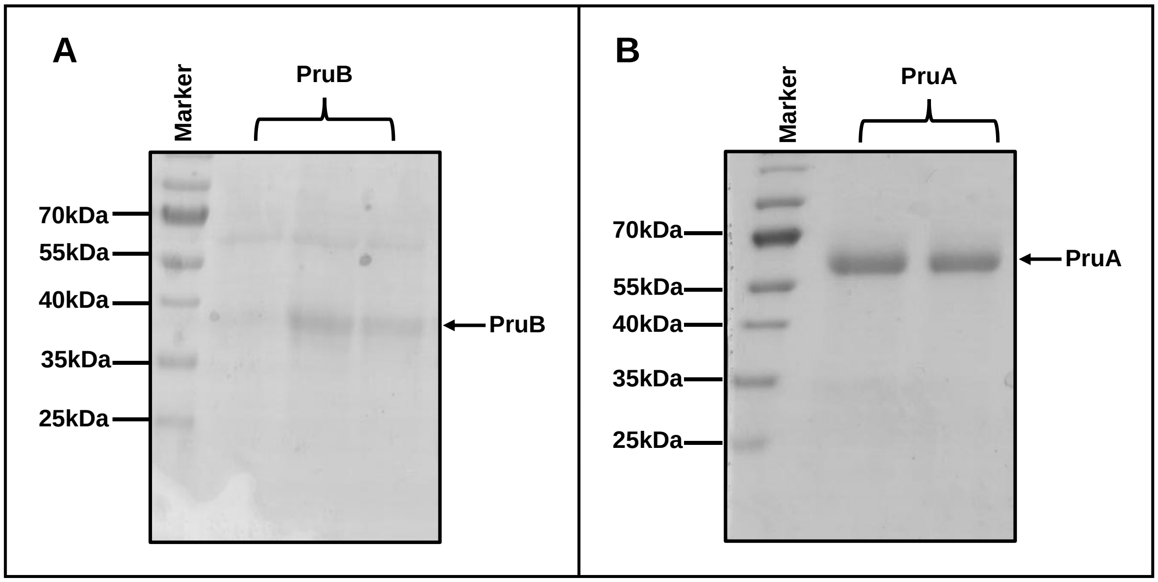

Figure S1. Purification of PruB and PruA. Representative 12%SDS-PAGE gels of 6His-PruB (Panel A) and 6His-PruA (Panel B) isolated from *M. smegmatis* after induction with 0.2% acetamide and purified on Ni-NTA affinity columns. Protein samples were electrophoresed on 12% polyacrylamide gels and stained with AcquaStain protein gel stain.

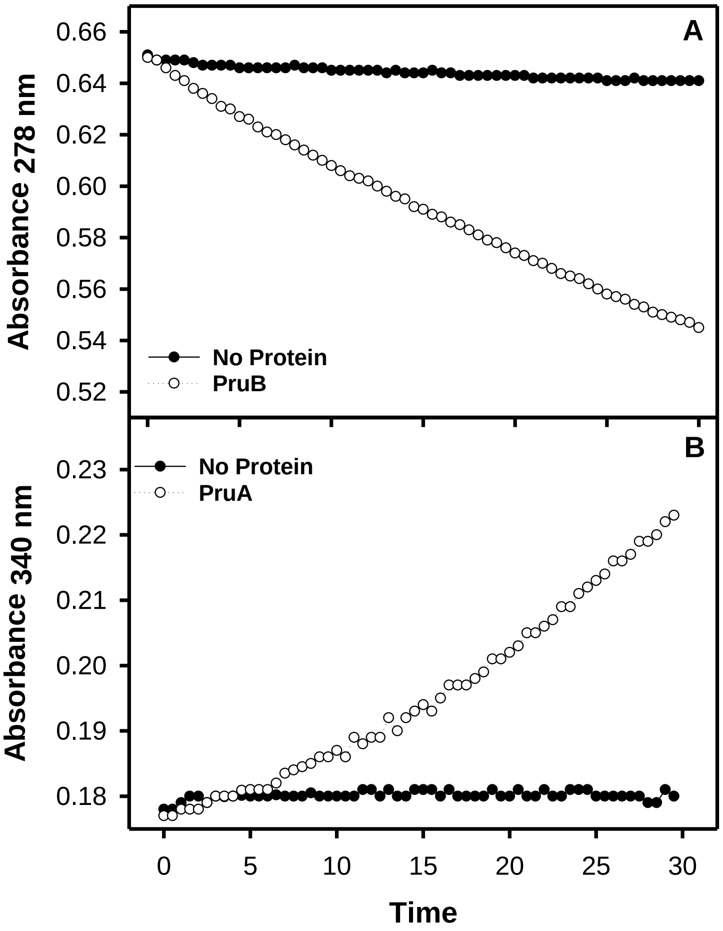

Figure S2. Representative UV-Vis traces of enzyme activity. For PruB (Panel A) enzyme activity was measured by monitoring decrease in absorbance at 278 nm. Assays contained 20mM Tris-HCl (pH 7.0), 25 ng of PruB, 20 mM L-proline, 100 μM UQ-1, 5 μM FAD in a 200 μL reaction volume. The initial reaction rate was calculated between 2-5 min. For PruA (Panel B) enzyme activity was measured by monitoring the formation of NADH at 340 nm. The initial reaction rate was calculated between 3 and 7 min. Assays contained 500 ng of PruA, 0.3 mM P5C, 0.2 mM NAD^+ in 200 μL of 20 mM Tris-HCl at pH 7.0. In both cases reactions were incubated at 25° C for 30 min.

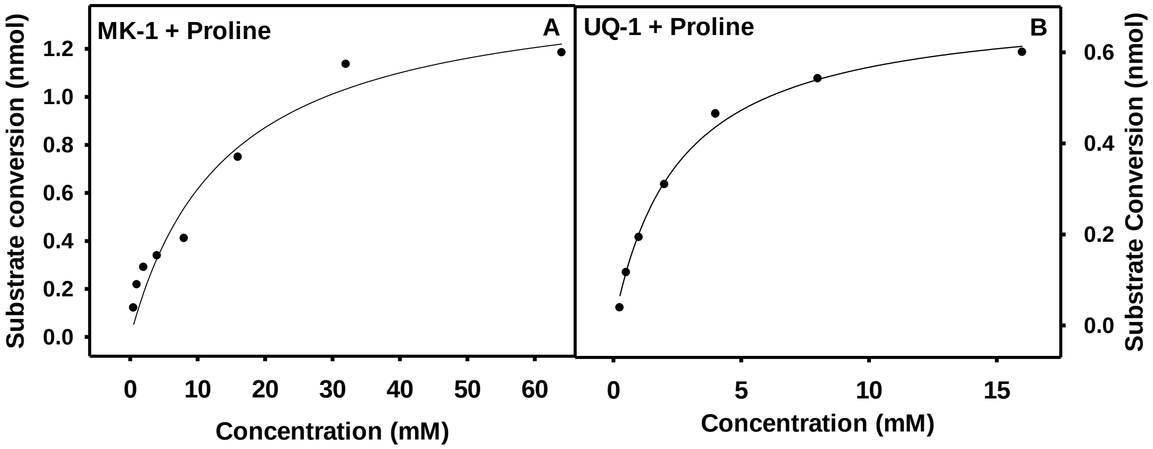

Figure S3: Representative Michaelis-Menten curves for PruB showing the effect of proline concentration in assays at saturating concentrations of MK-1 and varying concentrations of proline (Panel A) and the effect of proline concentration in assays at saturating concentrations of UQ-1 and varying concentrations of proline (Panel B). Assays contained 25 ng of PruB, in a final volume of 200 μL in 20 mM Tris-HCl pH 7.0 and were incubated at 25°C for 30 min. Activities were monitored via decrease in absorbance at 270 nm in the case of MK-1 and 278 nm in the case of UQ-1. Calculated kinetic parameters can be found in Table 2.

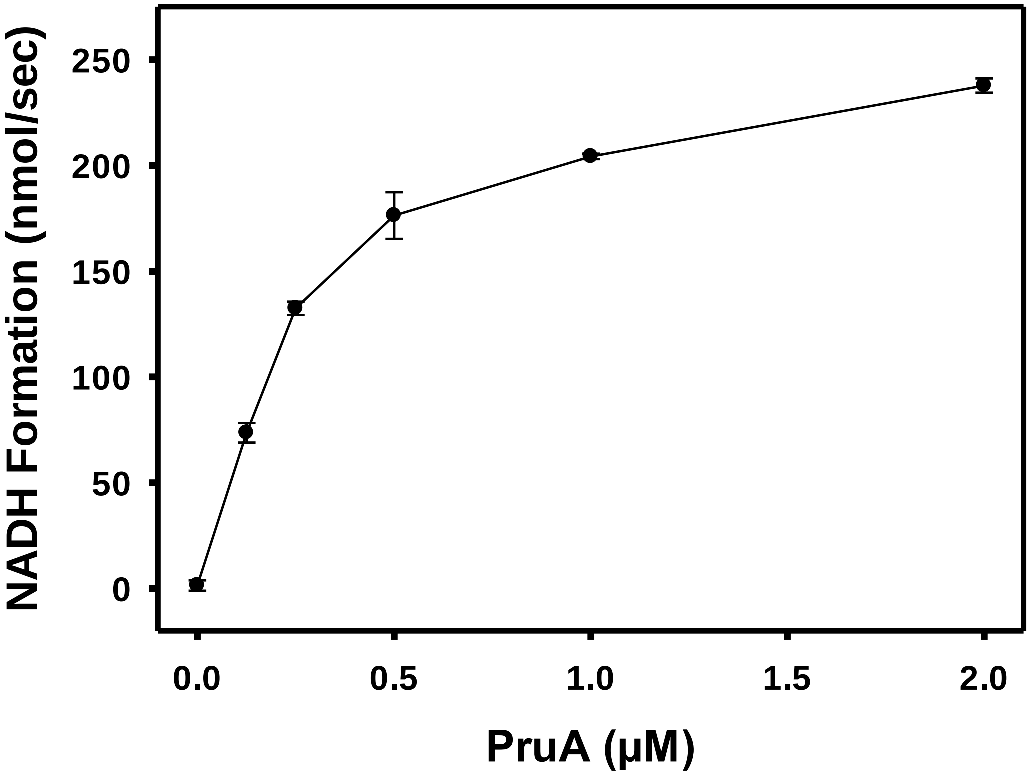

Figure S4: PruB-PruA coupled reaction: Reaction mixtures contained 20 mM Tris-HCl (pH 7.0), 5 μM FAD, 100 μM UQ-1, 200 μM NAD^+, 20 mM proline, and 0.5 μM PruB. The reaction was initiated by the addition of the indicated amount of PruA enzyme for 30 min. NADH formation was monitored at 340 nm. Error bars indicate the standard deviation of the mean of three independent experiments.

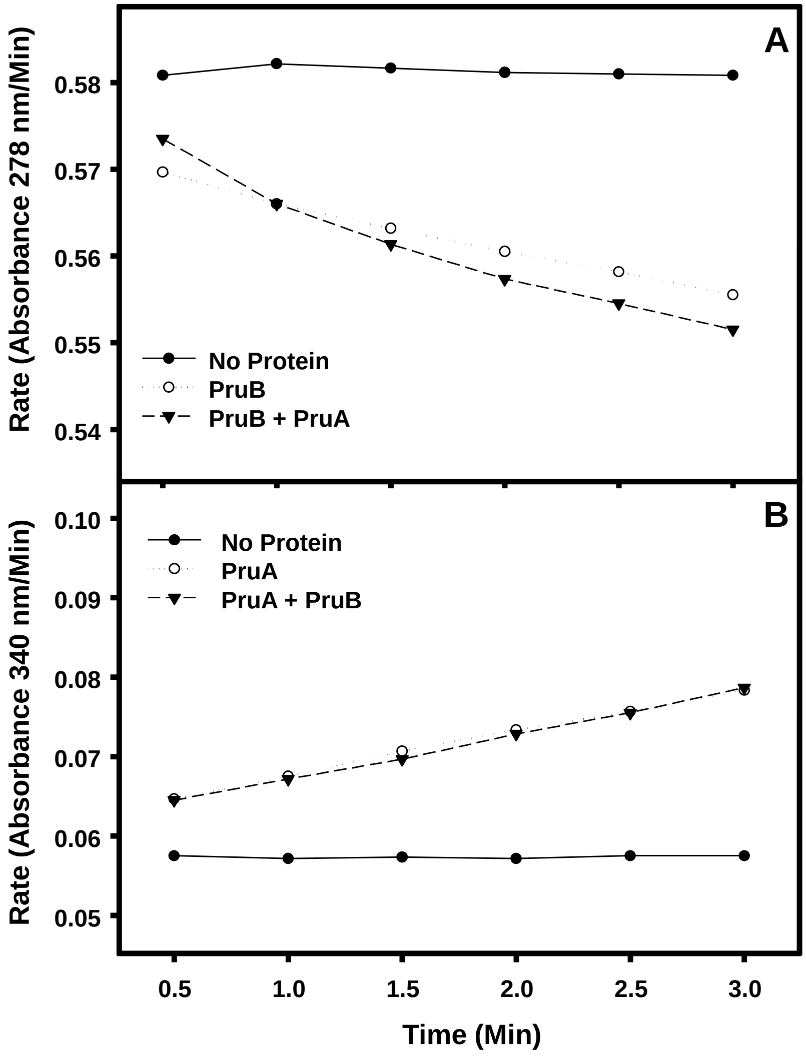

Figure S5: PruB and PruA do not affect the reaction rate of the other enzyme: Panel A - PruB activity was followed at 278 nm (UQ-1 reduction,). Assays

contained PruB (0.5 μM), an equimolar mixture PruB and PruA (0.5 μM each), or no protein, 20 mM proline, 5 μM FAD and100 μM UQ-1 in 200 μL of 20 mM Tris-HCl at 25°C. Panel B PruA activity was followed at 340 nm (NAD+ reduction). Assays contained PruA (0.5 μM), an equimolar mixture of mixture of PruA and PruB (0.5 μM each) or no protein, 300 μM DL-P5C and 200 μM NAD^+ in 200μL of 20 mM Tris-HCl at 25°C

Table S1. Primers used in the amplification of PruA and PruB constructs.

**Primer**	**sequences ('5----3')**	**Restriction Enzyme**
PruA Forward	'5- ATT cat atg GAC GCG ATC ACC CAG GTG CCG -3'	NdeI
PruA Reverse	'5- TAT aag ctt TCA GTC GAC CGC CAT GTG CGG-3'	HindIII
PruB Forward	'5- ATT cat atg GCC GGC TGG TTC GCG CAC-3'	NdeI
PruB Reverse	'5- TAT aag ctt TCA GCG CTC GGC GCA CCC-3'	HindIII

Table S2. Nonlinear fit of data shown in Figure 4 to a competitive inhibition model

Competitive (Full)
Number of Replicates: 3

Parameters

	Value	±Std. Error	95% Conf. Interval		
Vmax	0.5970	1.053e-2	0.5761	to	0.6179
Km	0.2326	1.399e-2	0.2048	to	0.2603
Ki	6.6651	0.5033	5.6672	to	7.6630

Goodness of Fit

Degrees of Freedom	105
AICc	-805.730
R^2	0.978
Sum of Squares	5.750e-2
Sy.x	2.340e-2
Runs Test p Value	0.221

Data

Number of x values	36
Number of replicates	3
Total number of values	108
Number of missing values	0

Enzyme Kinetics Data Summary

Competitive (Full)
Number of Replicates: 3

**[Substrate]**	**[Inhibitor]**	**Velocity**	**±Std.Err**	**Predicted**	**Max Residual**	Outliers
0.05	0.00	0.0910	3.5000e-3	0.1056	-2.1639e-2	
0.10	0.00	0.2030	7.0000e-3	0.1795	3.0486e-2	
0.20	0.00	0.2940	1.0500e-2	0.2760	2.8472e-2	
0.40	0.00	0.3990	1.0500e-2	0.3775	3.1987e-2	
0.80	0.00	0.4515	0.0210	0.4625	-5.3041e-2	
1.60	0.00	0.5005	3.5000e-3	0.5212	-2.7742e-2	
0.05	2.00	0.0665	7.0000e-3	8.4716e-2	-3.2216e-2	
0.10	2.00	0.1435	0.0140	0.1484	-3.2878e-2	
0.20	2.00	0.2450	2.4500e-2	0.2377	4.5817e-2	
0.40	2.00	0.3395	9.2601e-3	0.3400	0.0170	
0.80	2.00	0.4375	1.2619e-2	0.4333	2.8741e-2	
1.60	2.00	0.5075	7.0000e-3	0.5021	1.2380e-2	
0.05	4.00	0.0455	9.2601e-3	7.0711e-2	-3.9211e-2	
0.10	4.00	0.1330	3.5000e-3	0.1264	1.0054e-2	
0.20	4.00	0.1855	3.5000e-3	0.2087	-3.0191e-2	
0.40	4.00	0.2940	3.3753e-2	0.3093	-7.8273e-2	1
0.80	4.00	0.4305	0.0105	0.4075	3.3536e-2	
1.60	4.00	0.4760	0.0305	0.4844	-6.4352e-2	
0.05	8.00	0.0455	3.5000e-3	5.3141e-2	-1.1141e-2	
0.10	8.00	0.0875	0.0140	9.7595e-2	-2.4095e-2	
0.20	8.00	0.1505	3.5000e-3	0.1678	-2.0765e-2	
0.40	8.00	0.2310	2.1000e-2	0.2619	-5.1926e-2	
0.80	8.00	0.3535	9.2601e-3	0.3641	-2.8107e-2	
1.60	8.00	0.4445	1.5256e-2	0.4523	-3.2339e-2	
0.05	16.00	0.0315	0.0000	0.0355	-3.9994e-3	
0.10	16.00	0.0665	3.5000e-3	6.7014e-2	6.4859e-3	

0.20	16.00	0.1260	1.2124e-2	0.1205	2.6498e-2
0.40	16.00	0.2100	6.0622e-3	0.2005	1.9972e-2
0.80	16.00	0.3185	9.2601e-3	0.3002	3.5783e-2
1.60	16.00	0.4130	7.0000e-3	0.3995	2.0475e-2
0.05	32.00	0.0210	0.0000	2.1334e-2	-3.3438e-4
0.10	32.00	0.0455	3.5000e-3	4.1197e-2	1.1303e-2
0.20	32.00	0.0700	3.5000e-3	7.7075e-2	-1.4075e-2
0.40	32.00	0.1365	6.0622e-3	0.1365	-1.0524e-2
0.80	32.00	0.2170	9.2601e-3	0.2222	-2.2728e-2
1.60	32.00	0.3535	7.0000e-3	0.3239	4.3608e-2

Enzyme Kinetics Model Comparison

Study Type: Single Substrate - Single Inhibitor
Number of Replicates: 3

Rank by	Equation	R^2	AICc	Sy.x	Run Test	Convergence
1	$\dfrac{V_{max}}{[1+ (K_m/S)x(1+I/K_I)]}$ (Competitive)	0.97794	-805.730	2.340e-2	Pass	Yes
